# The economic burden of prostate cancer in Eswatini

**DOI:** 10.1186/s12913-022-07817-6

**Published:** 2022-04-11

**Authors:** Cebisile Ngcamphalala, Ellinor Östensson, Themba G. Ginindza

**Affiliations:** 1 Discipline of Public Health Medicine, School of Nursing and Public Health, University of KwaZulu-Natal, Mazisi Kunene Road, 4041 Durban, South Africa; 2 Department of Women’s and Children’s Health, Karolinska Institutet, Stockholm, Sweden; 3 Department of Medical Epidemiology and Biostatistics, Karolinska Institutet, Stockholm, Sweden

**Keywords:** Prostate cancer, Cost-of-illness, Eswatini, Premature mortality, Prostate antigen test

## Abstract

**Background:**

Prostate cancer is the fifth cause of cancer mortality among men worldwide. However, there is limited data on costs associated with prostate cancer in low- and middle-income countries particularly in the sub-Saharan region. From a societal perspective, this study aims to estimate the cost of prostate cancer in Eswatini.

**Methods:**

This prevalence–based cost-of-illness study used diagnosis specific data from national registries to estimate costs associated to prostate cancer during 2018. The prevalence-based approach was used employing both top down and bottom up costing approaches. Costs data included health care utilization, transport, sick leave days and premature death.

**Results:**

The total annual cost of prostate cancer was $6.2 million (ranging between $ 4.7 million and 7.8 million estimated with lower and upper bounds). Average cost-per patient for radiotherapy, chemotherapy and other non-medical direct costs (transport and lodging) were the highest cost drivers recording $16,648, $7,498 and $5,959 respectively whilst indirect costs including productive loss due to sick leave and pre-mature mortality was estimated at $58,320 and $113,760 respectively. Cost of managing prostate cancer increased with advanced disease and costs were highest for prostate cancer stages III and IV recording $1.1million, $1.9million respectively.

**Conclusions:**

Prostate cancer is a public health concern in Eswatini, and it imposes significant economic burden to the society. This finding point areas for policy makers to perform cost containment regarding therapeutic procedures for prostate cancer and the need for strategies to increase efficiencies in the health care systems for increased value for health care services.

**Supplementary information:**

The online version contains supplementary material available at 10.1186/s12913-022-07817-6.

## Background

Among cancers, prostate cancer is the third commonest cancer after breast and lung cancer and the fifth cause of cancer mortality among men [1, 2]. In 2018, the number of new cases increased from 1.1 million in 2012 to 1.3 million in 2018 accounting for about 7.1% of the total cancer cases globally and 15% among men [2]. The causes of prostate cancer is attributable to genetic and environmental factors [2]. However, the incidence and mortality rate vary substantially within and across regions. Notably, high-income countries (HICs) reports high incidence rate compared to low- and -middle income countries (LMICs) [2]. In contrast, mortality rate is higher in developing countries particularly in sub-Saharan Africa regions [3]. The inequalities observed across regions with respect to prostate cancer incidence and mortality are in part linked to availability of effective screening and improved treatment modalities which are directly linked to resources availability [3, 4]. In Eswatini, compared to other common cancers, prostate cancer is ranked third accounting for 7.6% of total new cases 1074 in 2018 [5].

Prostate cancer causes clinical and economic burden to patients and governments. Screening tests include prostate-specific antigen (PSA) and digital rectal examination (DRG) [6, 7]. A positive screening tests results indicate further investigation [6]. Whilst PSA is the frequent screening test, it has been argued that PSA could potentially cause harm by over diagnosing low risk cancers that otherwise would have remained without clinical consequences for life time if left untreated [8]. In turn, this increases costs for prostate cancer [9]. In Sweden, annual costs associated with prostate cancer (screening, diagnosis and treatment) was estimated at €281 million [9]. In Ontario, the mean per patient cost for prostate cancer–related medication was $1211 [10]. In Iran, the total annual cost of prostate cancer was estimated at $2900 million [11]. Other studies estimated the economic burden of prostate cancer along with other cancer type. A study focusing on European countries, ranked prostate cancer the fourth cancer disease to cause health care costs compared to lung (€18.8billion), breast cancer (€15 billion), colorectal cancer (€13.1 billion) [12]. Similarly, in Korea, prostate cancer was among the top four cancers attributing to economic burden of disease [13].

There is limited evidence on the economic burden of prostate cancer from LMICs. Estimation of the economic burden of disease provide insight on treatment modalities and associated costs. The study aims to investigate the societal cost of prostate cancer in Eswatini during 2018.

## Materials and methods

### Study area

Eswatini formerly known as Swaziland is a country in Southern African bordering South Africa and Mozambique with an estimated population of 1.2 million [14]. The country’s economy is tied to South Africa and Eswatini’s domestic currency (Lilangeni=SZL) is pegged at parity with South African currency (Rand=ZAR) such that Eswatini cannot conduct its own monetary policy [15]. Eswatini fiscal revenue largely depend on Southern African Customs Union (SACU) revenues and remittance flowing mainly from South Africa [16, 17]. SACU receipts account for about a third of Eswatini’s total revenue and grants. However, over the past decades, SACU revenues have consistently declined leaving Eswatini’s economy constrained. The country records high national level poverty rate and income inequality which does not commensurate with its middle-income status. The national poverty rate is 58.9% percent at the international $1.90 poverty line and Gini index- a measure of inequality is 49.3 [17]. Eswatini ranks near the bottom of the World Bank’s Human Capital Index, with a score of 0.37 in 2020. Eswatini health spending as a share of the total budget is estimated at 10.1% and health per capita is estimated at $ 248 per annum [16]. Whilst Eswatini’s health expenditure is comparatively higher to some other countries in the Southern African region, the country’s health outcomes do not reflect its spending levels on health and its middle-income status. The health care service delivery is made up of public and private health care. Compared to the public, the private health care systems is better equipped both infrastructural and human resources however, at high health care costs. As such, private health care is accessed by less than 10% of the population, mainly those who owns health insurance [18].

Diagnostic and treatment capacity of conditions including cancer remains limited in the country mostly in the public health system. Through a government funded scheme namely Phalala, the Eswatini citizens are supported to access specialized health care services from neighboring countries mainly South Africa.

## Methods of costing

This is a Cost of Illness (CoI) study investigating costs of prostate cancer from the societal perspective [19]. CoI studies estimate disease specific costs [20]. The prevalence based approach, was used employing both top down and bottom up costing approaches [19, 21]. The cost estimation involved identification, quantification and valuation of resources used. The total costs for prostate cancer was calculated by multiplying identified resources quantities and the respective unit costs. All costs were presented in US$ adjusted for 2018 ($1= SZL14.5).

### Study population

Data on prostate cancer prevalence and mortality in 2018 was obtained from the National cancer registry [14]. The National Cancer Control Unit is led by the Ministry of Health. To estimate direct non-medical costs and annual gross earnings, estimates were obtained from a previous study that collected data using a direct non-medical costs patient questionnaire from a previous study on women diagnosed with breast cancer and receiving follow-up care at Mbabane Government chemotherapy unit (outpatient) in 2018 [22].

## Management of prostate cancer in Eswatini

**Fig. 1:**
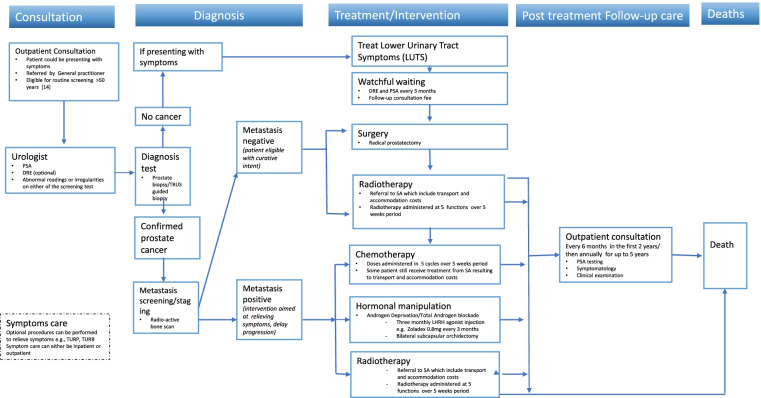
Simplified diagnosis and treatment pathway of patients diagnosed with prostate cancer. **PSA** Prostate specific antigen, **DRE** Digital rectal examination, **TURP** transurethral resection of prostate, **TURB** transurethral resection of bladder, **TRUS** Transrectal ultrasound, **LUTS** Lower Urinary Tract Symptoms

In Eswatini, routine prostate cancer screening is only recommended for men above age 50 every after two years [23]. The referral pathway shown in Fig. 1, simplifies the treatment pathway which begins by a man presenting with symptoms or eligible for screening at outpatient. Patient will be referred to urologist for screening tests including PSA and digital rectal examination (DRE) [6, 23]. These tests are not confirmatory however, they indicate changes in the prostate. Abnormal findings by either of the tests warrant further evaluation of patient and subsequent diagnostic test. These include biopsy (transrectal/perineal ultrasound guided biopsy (TRUS)). Patient with no cancer but presenting with symptom would receive management of lower urinary tract symptoms (LUTS). If cancer is confirmed further evaluation is conducted for cancer staging purposes in order to inform cancer management plan (metastasis screening). The evaluation includes radiology tests (bone scan, CT-scan and MRI pelvis). Staging is based on the tumor size (T) extent of lymph nodes involvement (N) and evidence of distant metastasis (M) [23, 24]. Depending on the risk score and prostate cancer stage, treatment include watchful waiting (cancer is monitored but not treated), surgery, radiation, chemotherapy and hormonal therapy (Androgen Deprivation Therapy) [23].

Most treatment modalities can be administered in various stages however for different intent [6, 23]. Radical prostatectomy, radiation and hormonal therapy can be applied for localised high risk prostate cancer (stage I and stage II) whilst for metastatic prostate cancer hormonal therapy will be first line in addition to radiotherapy, chemotherapy and hormonal therapy for palliation purposes. Radiation is not available in Eswatini and patient are referred to private hospitals in South Africa. Other surgical interventions for relieving symptoms such as transurethral resection of prostate (TURP) or bladder (TURB) can be conducted locally.

We used expert opinion from Mbabane Government Hospital - Chemotherapy Unit, Mbabane clinic -private hospital and information from Phalala Fund to establish patient referral pathway. Phalala Fund is a government funded scheme established to fund provision of specialized health care services to people of Eswatini that could not afford payment of specialized care that is not available in country [25]. The Eswatini standardized cancer care guidelines were used to establish screening, diagnosis and treatment variables. Costs were estimated based on market price. Radiotherapy is currently not available in Eswatini. As such, patients who require radiation are managed in South Africa through Phalala Fund. Chemotherapy is available locally through a government chemotherapy unit and local private clinic. However, it was established that most patients were still receiving chemotherapy from South Africa.

## Costs

From a societal perspective, costs associated with prostate cancer were estimated to assess economic burden of prostate cancer in Eswatini. Direct medical costs were divided into recurrent and capital costs [19]. Recurrent costs included personnel, travel, consumables including medical supplies, administration, utilities and overheads. Capital costs consistent mainly of equipment, building, vehicle and everything that have a useful life of more than one year. Costs for prostate cancer were determined based on the data source presented in Table 1. All costs were presented in US Dollars using 2018 average exchange rate (1 USD ($) = 14.5 SZL).

**Table 1:** Data variables and source for cost regarding screening, management and treatment of prostate cancer

Data	Data source	Price source
Estimated number of cases in 2018 = 90	Swaziland National Cancer Unit, Eswatini Prostate cancer cases in 2018	** N/A**
**Screening**		
Consultation fee	Mbabane Clinic	Private hospital
Prostate Specific Antigen (PSA)	Eswatini Health Laboratory Services	Private hospital
Digital rectal examination (DRE)	Interview with expert	Private hospital
**Diagnosis**		
TRUS guided Biopsy	Mbabane Clinic	Private hospital
Computed Tomography (CT scan)	Mbabane Clinic	Private hospital
MRI scan	Phalala fund	Private hospital
X-ray	Mbabane Clinic	Private hospital
Bone scan	Mbabane Clinic	Private hospital
**Intervention/Treatment**		
Watchful waiting (WW)	Interview with expert	Private hospital
Surgery	Mbabane clinic	Private hospital
Radiotherapy	Phalala Fund based on SA hospitals fees	Private hospital
Chemotherapy	Phalala Fund based on SA hospitals fees	Private hospital
Androgen deprivation	Phalala Fund based on SA hospitals fees	Market price
Hospitalization (local)	Phalala Fund based on SA hospitals fees	Private hospital
**Other direct costs**		
Transport and lodging costs in South Africa	Phalala Fund based on SA hospitals fees	Market price
Follow-up care (Year 1 following completion of treatment) Follow-up involves PSA testing, symptomology and clinical examination for metastatic cancer twice in a year	Based on reported prevalence	Private hospital

### Direct medical costs

Direct costs in this study include resource utilization for diagnosis, treatment (surgery, chemotherapy, radiotherapy and androgen deprivation therapy) and follow-up care. To estimate the directs costs, we estimated average cost of each intervention from screening, staging and treatment and multiplied by the number of corresponding patients who received the intervention. The number of men diagnosed with prostate cancer were obtained from the national cancer registry [26]. All diagnosed cases were assumed to have undergone screening test using PSA. Screening and diagnosis costs were obtained from private hospital and market pricing. Treatment costs mainly radiation, chemotherapy and androgen deprivation therapy were received from Phalala fund based on South African private hospitals fees. In Eswatini, a majority of the management costs are borne by the Eswatini Government through Phalala fund.

As per the standardized cancer care guidelines, we assumed that all the men with confirmed prostate cancer in 2018 underwent screening and diagnosis tests, treatment and incurred other direct costs including transport and accommodation. Follow-up care costs was estimated for one year for those reported alive in 2018.

### Direct non-medical costs

Transport cost including return was estimated based on required patient follow-up visits based on the Eswatini Standardized Cancer Care Guidelines which state that follow-up visit should be every six months for the first two years and annually for up to five years following surgery [23]. Transport cost was estimated based on data from a previous study on breast cancer women receiving follow-up care at Mbabane Government Cancer Unit [22]. We assumed that all men completed treatment in 2018 had follow-up visits as per the Eswatini Standardized Cancer Guidelines. This study estimated one-year follow-up costs.

### Indirect costs

We estimated the monitory value of prostate cancer related productivity loss due to morbidity (patient sick leave days incurred as a result of seeking health care) and pre-mature mortality).

The human capital method was used to estimate indirect costs related to productivity loss due to morbidity (sick leave as a result of seeking prostate cancer care) and pre-mature mortality [20]. We used average annual gross earnings computed from our previous study on breast cancer women receiving follow-up care in the chemotherapy unit, Mbabane Government hospital in Eswatini [22].

#### Morbidity costs

We estimated the number of sick leave days for men diagnosed with prostate cancer who are in the labor participation ages (18-60 years). Using findings from a previously published study [27], we assumed sick leave for an average of 54 days per person. The sick leave days included days for staging, treatment and follow-up care. Using findings from a previous study on breast cancer conducted in Eswatini [22], we assumed 20 working days per month and a full-time working day of 8 h with estimated costs per workday ($12) translating ($1.5) per work hour [22].

#### Mortality costs

To estimate the cost of lost productivity due to premature death related to prostate cancer, years of potential productive life lost (YPPLL) were calculated by subtracting age at death from the local retirement age of 60 years [28]. Prostate cancer age groups specific deaths were estimated assuming labor participation ages of Eswatini (18-60 years). We used full employment rate and annual average earnings obtained from a previous study. Average YPPLL was multiplied by average annual earnings. According to health economic recommendations, future costs were discounted at 3% and 5% [19, 29]. The number of prostate cancer related deaths was obtained from Eswatini Cancer Registry. In 2018, there were 31 prostate cancer related mortality with 4 that occurred within the labor participating ages of Eswatini (18-60) years [28].

## Cancer mortality and years of potential productive life lost (YPLL)

The number of prostate cancer related deaths was obtained from Eswatini Cancer Registry from which the years of productive life lost was calculated. In 2018, there were 31 prostate cancer related mortality out of which 4 occurred within the labor participating ages of Eswatini (18-60) years [28].

## Estimation of annual costs

We computed the aggregate total costs of screening, diagnosis and treatment of prostate cancer in 2018 as below:

$$Cost\;of\;disease\;=\sum\begin{pmatrix}Direct\;medical\;\cos ts\\+\\Direct\;non-medical\;\cos ts\\Direct\;\cos ts\end{pmatrix}+\begin{pmatrix}Morbidity\\+\\Mortality\\Indirect\;\cos ts\end{pmatrix}$$

Direct medical costs = Consisting of direct non-medical costs and direct medical costs.

Indirect costs = Consisting of morbidity costs and mortality costs (Patient time lost as a result of the condition and costs associated with premature mortality as a result).

All costs were reported in 2018 US dollars ($1=SZL14.5).

## Sensitivity analysis

Sensitivity analysis was performed using ± 25% to account for the cost of follow-up prevalent cancer cases and to account for unrecorded cases by the facilities.

## Results

### Directs costs

In 2018, there were 90 prostate cancer cases of which 89% aged 60 years an above. The average age was 73 years. Table 2, shows unit average costs for treating prostate cancer cases including other direct costs such as transportation and accommodation.

**Table 2:** Costs for screening, diagnosis and treatment of prostate cancer

Parameter	Variables included in the cost	Average (2018) USD
**Screening**	Consultation fee	41
	Prostate Specific Antigen (PSA)	16
	Digital rectal examination (DRE)	32
**Diagnosis**		
Pathology	TRUS guided Biopsy	147
Radiology	Computed Tomography (CT scan)- to rule out chest, abdomen and pelvis metastasis	862
	Magnetic resonance imaging scan (MRI)	1,034
	X-ray to rule out effusion	28
	Bone scan in locally advanced prostate cancer	607
	Ultrasound scan	103
**Treatment**		
Watchful Waiting (WW) cost include PSA test every three months and follow up consultation fee	PSA plus follow-up consultation fee	58
Surgery	Radical prostatectomy	5,726
	Orchiectomy	5,726
Radiotherapy	Administered at 5 function of a 5-week period	16,647
Chemotherapy (Brachytherapy or external beam radiation	Administered in 5 cycles over 5-week period	7,498
Androgen Deprivation Therapy (ADT)	Mostly Zoladex o.8 mg intramuscular (IM) for every 3 months	1,268
Symptoms relieving procedures (TURP/TURB)	Transurethral resection of the prostate or bladder (TURP/TURB)	5,872
**Other direct costs**		
Hospitalization costs (local)	Admitted for symptoms management procedures including transurethral resection of the prostate (TURP) and orchidectomy	5,872
Hospital admission in step down facility for late stage treatment (In South Africa)	Patient who require close monitoring following radiotherapy or surgery in hospital outside Eswatini	1,206
Transport and lodging cost in South Africa	All patients who received treatment in South Africa	5,959
Follow-up care (Year 1 following completing treatment) Follow-up is done using PSA testing, symptomology and clinical examination for metastatic cancer twice in a year	Follow up consultation, PSA tests.	94
**Total**		**58,796**

Cost distribution by disease stage is shown in Table 3. Following the Eswatini Standard Cancer Care Guidelines we assumed that all confirmed cases underwent similar screening, diagnosis and treatment pathway shown in Table 2, and simplified referral pathway shown in Fig. 1. The average costs for the different pathway including treatment intervention differed with the prostate cancer stage. Radical prostatectomy was more frequent with early stages of prostate cancer whilst interventions like chemotherapy were common with prostate cancer stages III and IV. Table 3 shows the prostate cancer costs distribution by stage.

**Table 3:** Costs for staging, management, and treatment of Prostate cancer stage I-IV

Staging and treatment variables	Unit cost ($)	I (T1)	II (T2)	III (T3)	1 V (T4)
Consultation for assessment	41	41	41	41	41
**Screening and diagnosis**					
Prostate Specific Antigen (PSA)	16	48	48	48	48
Digital rectal examination (DRE)					
TRUS guided Biopsy	147	147	147	147	147
MRI scan	1,034	1,034	1,034	1,034	1,034
Chest x-ray	28	27.6	27.6	27.6	27.6
Bone scan	607	607	607	607	607
Ultrasound	103	103	103	103	103
CT scan abdomen	862	862	862	862	862
**Treatment (Prostate Cancer prevalence in 2018=91 patient)**					
Watchful waiting (WW). Costs include PSA test every three months and follow up consultation fee	58	58	0	0	0
Radical prostatectomy	5,726	5,726	5,726	0	0
Orchiectomy (surgical castration)	5,726	5,726	5,726	5,726	5,726
Radiotherapy	16,648	16,648	16,648	16,648	16,648
Chemotherapy	7,498	0	0	7,498	7,498
Symptoms relieving procedures (TURP/TURB)	5,872	0	0	5,872	5,872
Other supportive drugs: Pain killers	60	60	60	60	60
Hormonal therapy (ADT) Zoladex 0.8 mg injectables	1,268	1,268	1,268	1,268	1,268
**Other costs**					
Hospitalization costs (local)	5,872	5,872	5,872	5,872	5,872
Hospital admission in step down facility for late stage treatment	1,206	1,206	1,206	1,206	1,206
Transport and lodging cost (in RSA)	5,959	5,959	5,959	5,959	5,959
Follow-up care (Year 1 following completing treatment)	94	94	94	94	94
**Total**	**58,824**	**45,486**	**45,428**	**53,072**	**53,072**

Radiation is not available in Eswatini and patients are referred to private hospitals in South Africa. On average, radiotherapy treatment is administered for a period of 5-weeks [25]. The estimated unit costs for radiotherapy was $16,648 whilst chemotherapy was $7,498. In addition to treatment costs, all patients referred for radiotherapy also incurred other direct costs including transport, lodging and allowance for accompanying staff (nurse and driver) at a unit costs $5,959, Table 3.

#### Direct non-medical costs

Using estimate from a previous study [22], the average transport cost per follow-up visit including return was $11 (inter quartile range (IQR)$4-46). On average, post treatment follow-up visits should be every 6 months resulting to four visits in a year including return. We assumed that all patients visited the hospital in the company of a relative. The total average transport costs including return was estimated at $5,029 (between $3,771 and 6,287 estimated with lower and upper bounds).

### Indirect costs

Productive loss due to sick leave as a result of patient seeking health care for prostate cancer was estimated at $58,320, Table 4. Out of the 90 patients diagnosed with prostate cancer, there were 13 men within the labor participating ages which were assumed to be on average sick leave of 54 days per person excluding short term sick leave of 14 days that is usually covered by employers. A total of 31 men died of prostate cancer in 2018 out of which 4 were less than 60 years. Costs due to prostate cancer premature mortality was estimated at $113,760, Table 5.

**Table 4:** Costs due to sick leave days associated with prostate cancer costs

	Numbers of sick leave days	Number of patients alive in 2018	Cost per workday ($)	Total productivity loss due to costs due to prostate cancer in 2018 ($) for all patient
Total patient	54	58	12	$37,584

**Table 5:** Mortality for prostate cancer

Mortality cost for Prostate cancer					
Age groups	Lost YPPLL (Average YPPLL for 1 patient in each age group)	Number of premature deaths before age 60	Average annual income	Mortality cost ($) with 3% Discount rate multiplied with the number of patients in this age group	Mortality cost ($) with 5% Discount rate multiplied with the number of patients in each age group
46-51	49	1	2690	29,632	10,676
52-56	54	3	2690	84,128	27,311
Totals	103	4	2690	113,760	37,987
YPPLL	211				
Average annual gross income = $2,690					

### Total annual costs

The total annual costs for prostate cancer was estimated at $ 6.2 million (between $4.7 million and 7.8 million estimated with lower and upper bounds), Table 6. Fourth 4% (40) of the cases were diagnoses with stage IV whilst only 11% (10) were diagnosed with stages I. Management of prostate cancer stages III and IV formed the greatest share of the costs for prostate cancer contributing about $1.2 and 2.1 million respectively. The total costs of stages I and II was estimated at $0.5 and $0.8 million. Transport and accommodation costs (cost incurred by those transferred to South Africa) were highest under other direct costs contributing about $0.5million. In 2018, there were 31 prostate cancer related deaths with only 4 occurred within the labor participating ages of Eswatini (18-60) years. The total year of productive life lost (YPPL) was 221 years. Indirect costs were estimated at $0.24 million and a majority (96%, $0.2 million) were productive loss from premature mortality, Table 6.

**Table 6:** Total Annual costs estimation for Prostate cancer (direct and indirect costs)

	Prevalence 2018	Cost per item ($)	Base case cost ($)	Range ($)	
**Parameter**	**Number**	**Average cost (2018)**	**Base costs (2018)**	**(Lower (-25%)**	**Higher (+25)**
**Direct costs (Health care costs**) consultation fee	90	41	3690	2768	4613
**Screening and diagnosis**					
Prostate Specific Antigen (PSA)	90	16	1448	1086	1810
Digital rectal examination (DRE)	8	0	0	0	0
TRUS guided Biopsy	90	147	13,213	9910	16,516
MRI scan	90	1034	93,060	69,795	116,325
Chest x-ray	90	28	2484	1863	3105
Bone scan	90	607	54,630	40,973	68,288
Ultra sound	90	103	9270	6953	11,588
CT scan abdomen	90	862	77,580	58,185	96,975
**Treatment**					
Stage I	10	45,486	454,861	341,146	568,577
Stage II	17	45,428	772,278	579,209	965,348
Stage III	23	53,072	1,220,659	915,494	1,525,824
Stage IV	40	53,072	2,122,886	1,592,164	2,653,607
**Other direct costs**					
Hospitalization costs (local)	90	210	18,900	14,175	23,625
Hospital admission in step down facility for late stage treatment	90	1206	108,540	81,405	135,675
Transport and lodging cost (in RSA)	90	5959	536,310	402,233	670,388
Follow-up care (Year 1 following completing treatment)	60	2662	159,720	119,790	199,650
**Total direct**		209,892	5,645,839	4,234,379	7,057,299
**Direct non-medical cost**					
Transport costs for follow-up visits ,patient	59	2513	148,267	111,200	185,334
Transport costs for follow-up visits, accompanying relative	59	2513	148,267	111,200	185,334
**Total Direct non-medical costs**		5026	296,534	222,401	370,668
**Indirect costs**					
Morbidity costs due to sick leave	13	648	8424	6318	10,530
Premature mortality costs	4	57,675	230,700	173,025	288,375
**Total indirect costs**		58,323	239,124	179,343	298,905
**Total**		**268,215**	**6,181,497**	**4,636,123**	**7,726,871**

## Discussion

The current study assessed the costs associated with prostate cancer in Eswatini, that is, screening, diagnosis, treatment and follow-up care. The study considered direct costs including follow-up care costs within one year of diagnosis. To our knowledge this is the first study to estimate the economic burden of prostate cancer in Eswatini. The estimated annual prostate cancer burden was $ 6.1 million in 2018. About 89% of the patient aged 60 years and above. Given the Eswatini Standardized Cancer Care and Guidelines [21], we assumed that all patients diagnosed in 2018 underwent the screening and diagnostic procedures. Treatment costs varied by cancer stage reflecting the utilization of treatment modalities per stage hence high costs observed in stages III ($1.2million) and IV (2.1million) versus Stage I and II with $0.5 and $0.8 million respectively. The findings indicate that managing advanced stages of the disease increases health care costs.

The study findings were in accordance with findings from other studies. A study assessing health care costs associated with prostate cancer in Canada reported increasing costs per stage I ($1,297), II ($3,289), III ($1,495), IV ($5,629) and V ($16,020) [30]. Similarly, a study conducted in Iran concluded that health care costs for metastatic stages were the highest compared to treatment costs for localized prostate cancer [11]. More studies had similar conclusions [31, 32]. Slightly different findings were from the United State of America who reported high treatment costs for initial diagnosis and metastatic phase with radical prostatectomy being the main cost driver [33]. Whilst in this study we found lesser cost with early stage cancer, however, both studies observed increasing costs with advanced cancer stages. Also, the differences could be partly explained by the men (20%) diagnosed with early stages of prostate cancer in our study. A systematic review of registry-based studies assessing economic burden of prostate cancer in Europe found that cost distribution across prostate cancer stages varied across countries [34]. This can be attributed to differences in prostate cancer detection and country specific management practice [34]. The authors also acknowledged the difference in methodologies applied in the studies as possible explanation to the varying outcome observed.

There seems to be lack of global consensus on prevention strategies particularly age of screening. The United State Preventive Service Task Force (USPSTF) recommend against routine screening for men 70 years and older for prostate cancer particularly using prostate specific antigen screening [35]. The Eswatini Standardized Cancer Care and Guidelines also discourages routine prostate cancer screening with an exception for men 50 years and above or symptomatic [23]. Other studies argue that increased screening lead to increased detection of low-grade cancers resulting to patient with indolent tumors receiving aggressive treatment [36].

In LICs such as Eswatini, the challenge is likely to be on a different direction than over diagnosing and consequently overt treatment. Lack of screening and comprehensive treatment remains the greatest challenge for most LMICs and LICs. Eswatini is not different from other low middle income countries from whom late diagnosis coupled with limited treatment options remains a challenge. In Eswatini, in 2018, more than 80% of the patients were diagnosed with advanced cancer (stages III and IV), yet major treatment is not available in country. These include radiotherapy and androgen deprivation therapy (ADT). Accessing care outside the country comes with additional costs, mainly accommodation, transportation and meals for patients referred to South Africa.

Lack of specialized and costly care have been reported in other countries particularly in Africa and mortality from prostate cancer is the highest in these countries and there is lack of cancer treatment guidelines [4, 37].

There is an urgent need to strengthen health systems enablers [38]. These include investments in the establishment of local cancer treatment centers, optimizing health workforce competencies throughout the continuum of care and ensuring availability of medical products and diagnostics technologies to facilitate local diagnosis, staging and management.

Despite the evidence that prostate cancer is a major public health challenge, literature on the economic burden of prostate cancer is however limited and severely so in low income countries particularly in the sub-Saharan region. Findings from a systematic review on the costs of prostate cancer studies indicated a need not only for harmonized methodologies but also to expand research in this field [39]. Similarly, another systematic literature review of registry-based studies reached similar conclusion on the need for further research in cost of illness studies focusing on prostate cancer [40].

In the study we assessed indirect costs by estimating the costs associated with unpaid sick leave days and productive loss due to premature mortality from prostate cancer. Of the total costs, indirect costs accounted for 4.2% ($0.24 million). Comparing these findings to previous cost analysis studies for prostate cancer, most of the studies did not consider assessing indirect costs, however a study from Sweden reported low proportion of productivity loss associated with prostater cancer [9]. Further comparison of the findings with studies from other cancer types conducted in Eswatini [22, 41], the indirect costs from this study accounted for a lesser share of the total cost. This could partly be explained by the fact that most participants (89%) were above the labor participating ages (18-60 years) and few deaths occurred below age 60 years. A similar pattern was observed in Sweden, again the finding were attributed to low number of prostate cancer cases and deaths among labor participation groups [9].

The key strength of our study was that this is the first study to estimate cost associated with prostate cancer in Eswatini. The study considered both direct and indirect costs of prostate cancer. Our study has notable findings that has implications on health care systems strengthening and resources allocation in Eswatini. Our study present description of resource utilization and associated health care costs in managing prostate cancer in Eswatini.

An important limitation is the absence of index cost in Eswatini. We considered private and market prices for best possible price estimates.

The estimates presented were based on available data however, estimates could be conservative due to several reasons, First, due to limited data availability we used information from literature and interview with experts for some treatment variables, as such, some information can be subject to context and preferences. Secondly, we only considered costs in the first year of diagnosis yet cost for follow-up care can be even beyond five years [6, 42]. Lastly, we employed human capital approach to estimate the costs related to productivity loss associated with prostate cancer. Whilst this is a commonly applied approach, it is mostly criticized for excluding individuals above the labor participation age group yet there is argument that some of those people can still be involved in labor activities that gives meaningful income. Another author argues that this has severe implication when valuing productivity loss for prostate cancer given that a majority of the patients are diagnosed after they have past the retirement age.

## Conclusions

The findings of the study indicated that costs attributed to prostate cancer were substantial and they are a public health concern. The findings were consistent with those of other countries, a majority of which were conducted in developed countries. The study demonstrated the interventions and associated costs. Radiotherapy was the most expensive treatment intervention in Eswatini, yet other studies cited surgery related intervention as the major costs driver. This is a reasonable finding in the context of Eswatini given that radiotherapy treatment is not available locally, patients are referred to private hospitals outside the country. The findings point areas for policy makers to perform cost containment regarding therapeutic procedures for prostate cancer. Also, the study findings demonstrate that prostate cancer costs are likely to increase in future and there is a need for strengthening adherence to the Eswatini Standardized Cancer Care and Guidelines in order to ensure that resources are invested to diagnosing the most at risk groups.

## Supplementary Information

**Additional file 1.** Direct non-medical costs patient questionnaires.pdf

## Data Availability

All data generated or analyzed during this study are included in this published article. See study tables and figures.

## Abbreviations

ADT: Androgen Deprivation Therapy
DRE: Digital Rectal Examination
PSA: Prostate Specific Antigen test
TRUS: Transrectal ultrasound
LUTS: Low Urinary Symptom
HICs: High Income Counties
LICs: Low Income Countries
LMICs: Low Middle Income Countries
SDGs: Sustainable Development Goals
