# Supplementary material for: The economic burden of prostate cancer in Eswatini
Source: BMC Health Serv Res. 2022 Apr 11;22:483. doi: 10.1186/s12913-022-07817-6 (PMC9004055; doi:10.1186/s12913-022-07817-6)
Supplement: Supplementary file 1 — Additional file 1. Direct non-medical costs patient questionnaires.pdf [file 12913_2022_7817_MOESM1_ESM.pdf]

THE ECONOMIC BURDEN OF CANCER IN THE KINGDOM OF ESWATINI

Cost of Illness study

Direct non-medical costs patient questionnaires

NHRRB Approval No.:

Questionnaire No. (Study ID) [ ][ ][ ]

Date: [ ][ ] / [ ][ ] / [ ][ ][ ][ ]
dd mm yyyy

Instructions/Summary

1. This questionnaire is for collecting direct non-medical costs incurred by the patient as a result of seeking health care services following confirmed diagnosis with either of the following cancers: 1). CERVICAL 2). BREAST and 3). PROSTATE. These include cost distance, time and travel related costs.
2. Data from patients with NO CONFIRMED DIAGNOSIS of cervical, breast and prostate cancer MUST NOT be collected

Part 1: The following are questions about your background

Please mark or fill in the answer to each question.

No.	Questions
1.	Date of birth \........\........\.............. Dd \ mm\ yy
2.	**Marital status**<br>1. Single (never been married)<br>2. Living with partner (cohabiting)<br>3. Married<br>4. Divorced/separated<br>5. Widow<br>6. Other (please specify):………………………………………………….
3.	**What is your level of education**<br>1. Never been to school<br>2. Primary<br>3. Secondary/High school<br>4. Tertiary (college/university)
4.	**What is your employment status/ main activity (*more than one answer is possible*)**<br>1. Gainfully employed<br>2. Self-employed<br>3. Studying<br>4. On sick leave<br>5. retired<br>6. Other (please specify):…………………………………………………
5.	**How many hours per week are you involved in the above activities (including overtime work)?**<br>State the **time you are (working /studying) in whole hours ……………………………(e.g.: 40hours)**<br><br>**State the time you are on sick leave in days …………………………………………(**e.g.: 30 days)
6.	**What is your gross annual income? Income refers to salary, pension, student aid, and compensation from insurance fund, income from business or farm over the entire year.**<br>1. 1 - 19000SZL<br>2. 20 - 39 000SZL<br>3. 40 - 59 000 SZL<br>4. 60-79 000 SZL<br>5. 80-99 000 SZL<br>6. 100- 199 000 SZL<br>7. 200-299 000 SZL<br>8. 300-399 000 SZL<br>9. 400 -499 000 SZL<br>10. 500 - 599 000 SZL<br>11. 600 -699 000 SZL<br>12. 700 – 799 000 SZL<br>13. 800 – 899 000 SZL<br>14. 900 – 999 000<br>15. 1 – 1.9 million SZL15.280-299 thousand kronor<br>16. 2 million or more

Part 2. The following are questions about the time and travel related to follow-up care post diagnosis

Please mark or fill in the answer to each question.

No.	Questions

1.	**Which means of transportation did you use to get here? If more than one, please specify the main one.**<br>1. By foot<br>2. Public transport (bus/kombis)<br>3. Car as a driver<br>4. Car as passenger<br>5. Taxi<br>6. Other (please specify): …………………………………
2.	**From where did you travel to come to the clinic/hospital/facility for the care following your cancer diagnosis?**<br>1. Your home<br>2. School/Training<br>3. Your workplace<br>4. . Other (please specify):……………………………………………………………….
3.	**Did you do any other errand/business/activity on your way here?**<br>1. Yes 2. No (If **No** skip **Q4** to **Q5**)
4.	**What was the errand/business/activity which you did on the way here?**<br>1. Work<br>2. School<br>3. Shopping<br>4. Service (bank, post office, etc.) 9.<br>5. Recreation/leisure activity<br>6. Visit family and/or friends<br>7. Pick up/drop off your child(ren)<br>8. Other private errand/activity
5.	**About how long in minutes did it take you to travel to the clinic**? *Exclude the time for any other errands* …………………..minutes
6.	**What is the approximate distance in kilometers between the place/location from which you traveled and the clinic?** ………………. kilometers
7.	**Where will you go after your visit to the clinic?**<br>1. Your home<br>2. Your workplace<br>3. School/Training<br>4. Other (please specify)……………………………………………………………….
8.	**Which means of transportation will you take to return to your specified location?**<br>*If more than one, please specify the main one.*<br>1. By foot<br>2. Bicycle<br>3. Car as driver<br>4. Car as a passenger<br>5. Local/regional bus<br>6. Taxi<br>7. Other, (please specify):……………………………………………….
9.	**Approximately how long in minutes do you estimate it will take to travel to your destination after the clinic visit?** ………….minutes
10.	**Approximately how far in kilometers do you estimate it is to return to your destination after the clinic visit?** ………….kilometers
11.	*Answer Q11-F211-F213 only if you traveled mainly by car*.<br>**About how much do you estimate that you paid to travel here? Include the cost per kilometer by car.** SZL ……………………… (*Estimate one way costs*)
12.	**Did you need to cancel or postpone some activity in order to come to the facility for your care**?<br>1. Yes 2. No **–** If no skip **Q.13** to **Q.14**

13.	**What was the activity which you needed to cancel or postpone?**<br>1. Work-related<br>2. Visit family and/or friends<br>3. School-related 7. Pick up/drop off child(ren)<br>4. Shopping 8. Pick up/drop off another person<br>5. Recreation/leisure activity<br>6. Other private errand/activity
14.	**Did you take time off from work to come to the seek care related to your disease**?<br>1. Yes 2. No (If no skip **Q.15** to **Q.16**)
15.	**How many hours did you take off from work?** ……………hours
16.	**Did you need help from another person to be able to come to the facility for cancer related service** (for example, child care or other support)?<br>1. Yes 2. No (If no skip **Q.17** to **Q.18**)
17.	**What is your relationship to that other person?**<br>1. Partner<br>2. Relative<br>3. Friend<br>4. Child care provider service-**Go directly to Q21**<br>5. Other (please specify)………………<br>6. (999) Don't know/Prefer not to answer
18.	**Did that person have to take time off from work?**<br>1. Yes 2. No 999. Don't know
19.	**About how many hours do you estimate that this person took off from work?**<br>……………hours
20.	**What is that person's annual income before taxes?** Income refers to salary, pension, student aid, compensation from insurance fund, income from own business or farm.<br>1. 1 - 19000SZL<br>2. 20 - 39 000SZL<br>3. 40 - 59 000 SZL<br>4. 60-79 000 SZL<br>5. 80-99 000 SZL<br>6. 100- 199 000 SZL<br>7. 200-299 000 SZL<br>8. 300-399 000 SZL<br>9. 400 -499 000 SZL<br>10. 500 - 599 000 SZL<br>11. 600 -699 000 SZL<br>12. 700 – 799 000 SZL<br>13. 800 – 899 000 SZL<br>14. 900 – 999 000<br>15. 1 – 1.9 million SZL15.280-299 thousand kronor<br>16. 2 million or more
21.	**For approximately how many hours did you need childcare?** ……………hours
22.	**Approximately how much do you pay per hour for childcare service?**<br>……………SZL per hour

Name of data collector: ……………………………………….
Date: ………………………………………………………………
Signature:………………………………………………………….

Participant's signature:…………………………………….

Date:……………………………………………………………….
